# Role of rhBMP-7, Fibronectin, And Type I Collagen in Dental Implant Osseointegration Process: An Initial Pilot Study on Minipig Animals

**DOI:** 10.3390/ma14092185

**Published:** 2021-04-24

**Authors:** Gianmario Schierano, Rosa Angela Canuto, Mitzy Mauthe von Degerfeld, Roberto Navone, Bruno Peirone, Giulio Preti, Giuliana Muzio

**Affiliations:** 1 Department of Surgical Science, C.I.R. Dental School, University of Torino, Via Nizza 230, 10126 Torino, Italy; giulio.preti@unito.it; 2 Department of Clinical and Biological Sciences, University of Torino, Corso Raffaello 30, 10125 Torino, Italy; rosangela.canuto@unito.it (R.A.C.); giuliana.muzio@unito.it (G.M.); 3 Department of Veterinary Sciences, University of Torino, Largo Paolo Braccini 2, Grugliasco, 10095 Torino, Italy; mitzy.mauthe@unito.it (M.M.v.D.); bruno.peirone@unito.it (B.P.); 4 Department of Medical Science, University of Torino, Via Santena 5, 10126 Torino, Italy; roberto.navone@unito.it

**Keywords:** dental implant, fibronectin, rhBMP-7, type 1 collagen, cytokines, bone morphogenetic proteins

## Abstract

Background: The biological factors involved in dental implant osseointegration need to be investigated to improve implant success. Methods: Twenty-four implants were inserted into the tibias of six minipigs. Bone samples were obtained at 7, 14, and 56 days. Biomolecular analyses evaluated mRNA of BMP-4, -7, Transforming Growth Factor-β2, Interleukin-1β, and Osteocalcin in sites treated with rhBMP-7, Type 1 Collagen, or Fibronectin (FN). Inflammation and osteogenesis were evaluated by histological analyses. Results: At 7 and 14 days, BMP-4 and BMP-7 increased in the sites prepared with rhBMP-7 and FN. BMP-7 remained greater at 56 days in rhBMP-7 and FN sites. BPM-4 at 7 and 14 days increased in Type 1 Collagen sites; BMP-7 increased from day 14. FN increased the TGF-β2 at all experimental times, whilst the rhBMP-7 only did so up to 7 days. IL-1β increased only in collagen-treated sites from 14 days. Osteocalcin was high in FN-treated sites. Neutrophilic granulocytes characterized the inflammatory infiltrate at 7 days, and mononuclear cells at 14 and 56 days. Conclusions: This initial pilot study, in a novel way, evidenced that Type 1 Collagen induced inflammation and did not stimulate bone production; conversely FN or rhBMP-7 showed neo-osteogenetic and anti-inflammatory properties when directly added into implant bone site.

## 1. Introduction

To improve the peri-implant osteogenesis and the bone healing time, the biological factors involved in these processes must be evaluated. It is known that the porous implant surface (dual etching, sandblasting and etching, anodized) stimulates peri-implant osteogenesis to a greater extent than the smooth implant surface [1].

Indeed, levels of biological factors, such as bone morphogenetic protein BMP-4 and transforming growth factor TGF-β, increased earlier around porous implants, in a study in which the bone formation was evaluated by histological analysis [1].

Moreover, new surgery devices are currently used to improve osseointegration process [2,3,4,5,6].

Generally, BMPs regulate osteoblast and chondrocyte differentiation during skeletal development and healing of bone fractures [7,8,9,10].

The BMP-4, a member of the BMP family, shows bone-inducing properties.

Based on some studies, TGF-β stimulates in vivo [11,12] osteoblast precursors proliferation rather than osteoblastic differentiation [13,14].

Thus, it may be postulated that TGF-β could stimulate BMPs expression in the early phases of bone healing, just before the BMPs exert their effects.

Different substances placed around titanium (Ti) implants have been studied to promote bone formation.

In a previous study, nanoporous Ti implants bearing a covalently linked surface hyaluronan (HA) layer were used for in vivo evaluations in rabbits.

The HA-coated implants showed a high level of osseointegration in marrow-rich trabecular bone [15].

In a similar way, it has been reported that osseointegration is favored by calcium phosphate coatings of implants and by the presence of collagen [16].

Moreover, in studies on bone synthesis by using scaffolds, the addition of BMP-7 and BMP-2 improved bone production [17,18].

Based on the above observations, the purpose of this initial pilot study was to compare the osseointegration process of porous implants in the presence or absence of Fibronectin (FN), rhBMP-7, or Type 1 Collagen in minipigs.

## 2. Materials and Methods

### 2.1. Animals

The allocation sequence was adequately generated and applied, as follows. Six healthy male adult minipigs, weighing 70 to 80 kg, at the end of their growth (about three years old), were utilized in this study. The animals were randomly selected from a group of similar minipigs by a veterinarian (dedicated veterinary to animal logistics) who did not participate in the research. In particular, the six animals used in the study belonged to a group of 15, all having the same characteristics in terms of sex, age, weight, and health status, with health evaluated by blood analyses. For the random selection, each animal was marked with a number and six numbers were drawn corresponding to the six animals used in the experiment. The same procedure was used for the assignment of the 6 animals to the two experimental groups.

Each animal was placed in a single dedicated box in the Animal Pathology Department.

All boxes were located in the same dedicated housing room, with the same humidity and temperature. The minipigs were fed standard pelleted cereal food and were given water ad libitum.

The concept of the blind study was maintained for all experimental phase times.

This study was approved by the appropriate Bioethics Committee of the University of Torino, Italy, with resolution dated 26 February 2003, project title “Histological, immunohistochemical, radiological and biomolecular analysis of the titanium dental implant interface and bone” and recertified from the same Bioethics Committee with protocol number 58539 dated 2 February 2021.

### 2.2. Implant Insertion

Twenty-four titanium implants (TiUnite MKIII 3.75 × 10 Nobel Biocare Italiana, Agrate Brianza, Milan, Italy) were surgically inserted into the tibias of the six minipigs.

For this, the six animals were divided randomly into two groups of three each and underwent surgery. After pre-anesthetic sedation with 2% xylazine (Rompun 2%, 2.3 mg/kg, Bayer S.p.A., Milan, Italy) (2.3 mg/kg) and tiletamine/zolazepam (Zoletil 100-Virbac 20%, 6.3 mg/kg, Laboratoires Virbac, Carros, France) (6.3 mg/kg), surgery was performed under intubation anesthesia with isoflurane/halothane and O_2_.

**Figure 1:**
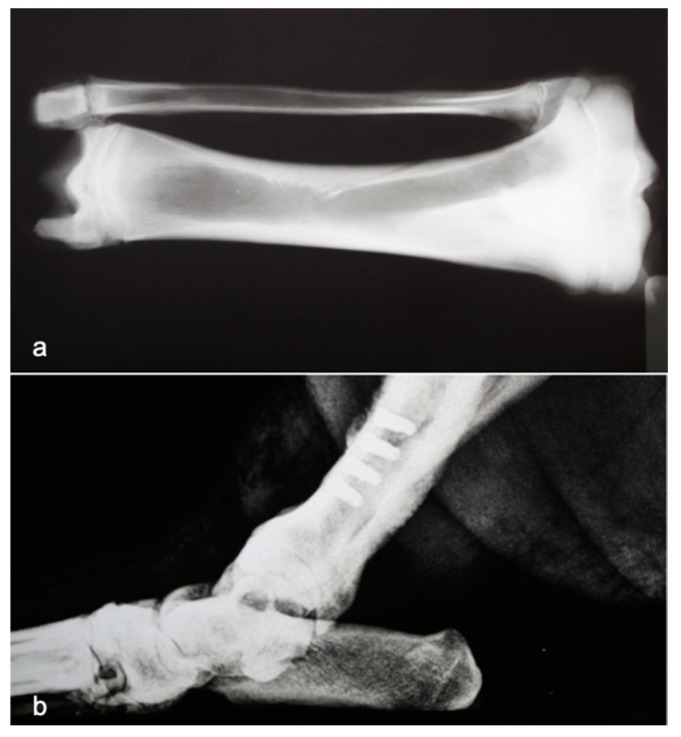
X-ray tibia of minipig showing the relationship between cortical and cancellous bone (**a**). X-ray tibia with implants (**b**).

The right hind leg was prepared in a standard sterile fashion. The tibia was exposed, and the implant was inserted using a 45 N·cm torque by using the drilling technique according to the Brånemark protocol [19] (Figure 1).

**Figure 2:**
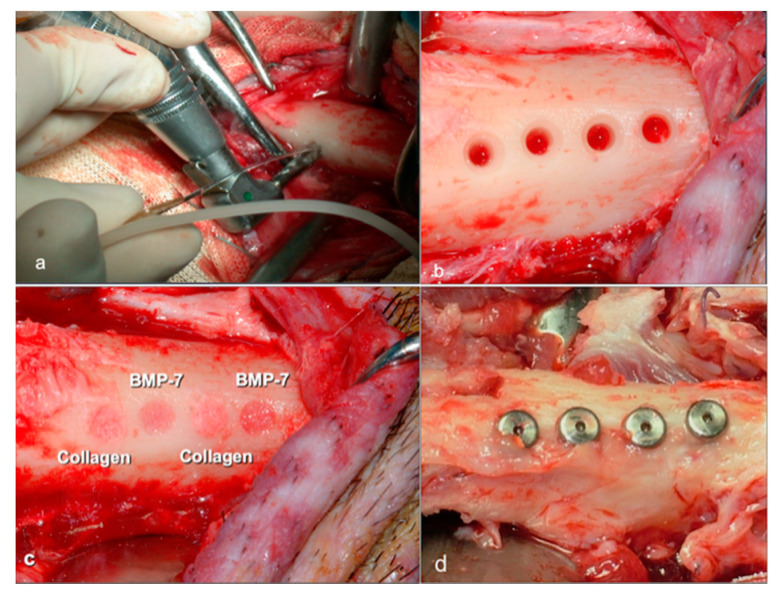
Example of implant site preparation: (**a**,**b**), treated with rhBMP-7 or collagen (**c**). Implant sites, at 56 days, before sliced dissection (**d**).

In the first group, each animal received two implants in two sites, with rhBMP-7; in the other two sites, two implants with Type 1 Collagen were inserted, resulting in a total of 12 implants in the three animals (6 with rhBMP-7 and 6 with Type 1 Collagen). rhBMP-7 was prepared as 3.5 mg mixed with Type 1 collagen to have 1 g of substance and reconstituted with 3 mL of sterile saline solution, corresponding to 1.16 mg/mL (Stryker BIOTECH, MI, USA); Type 1 Collagen as 333.3 mg/mL of sterile saline solution (SIGMA-Aldrich Corporate, St. Louis, MO, USA). Collagen Type 1 was used as a carrier of the rhBMP-7 (Figure 2).

**Figure 3:**
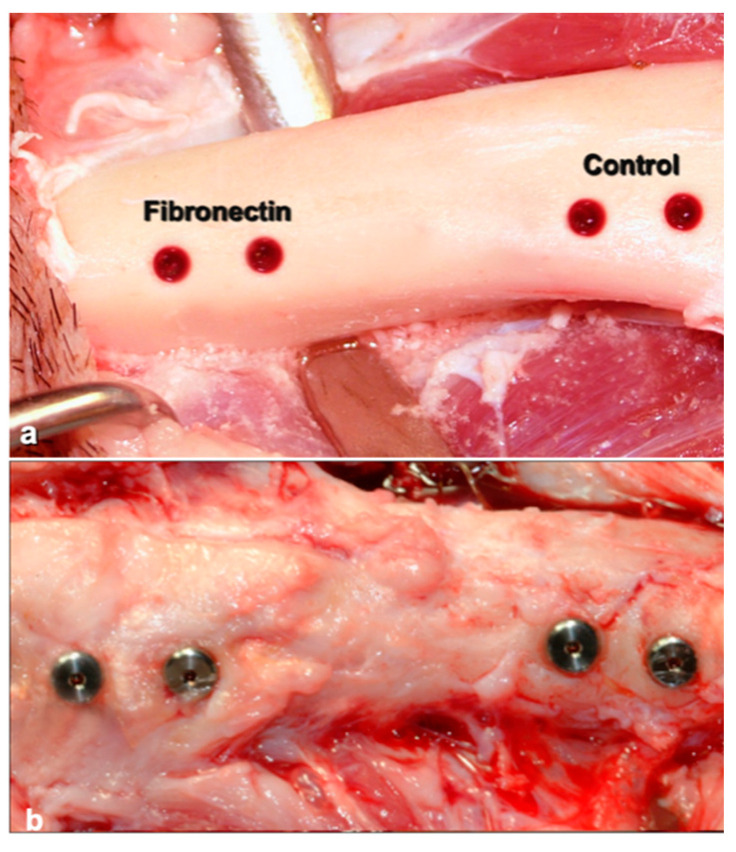
Example of implant site preparation: Untreated (control) or treated with Fibronectin (**a**). Implant sites, at 14 days, before sliced dissection (**b**).

In the second group, each animal, in two sites, received two implants with FN and, in two other sites, two implants without substance addition (control implants), resulting in a total of 12 implants (6 with FN and 6 Control). FN was prepared as 16.6 mg/mL (GIBCO Invitrogen, Life technologies corporation, Carlsbad, CA, USA) (Figure 3).

rhBMP-7, Type 1 Collagen and FN were directly positioned in the osteotomy sites.

To determine baseline (time 0) values, tibial bone specimens were collected during the surgery stage.

**Figure 4:**
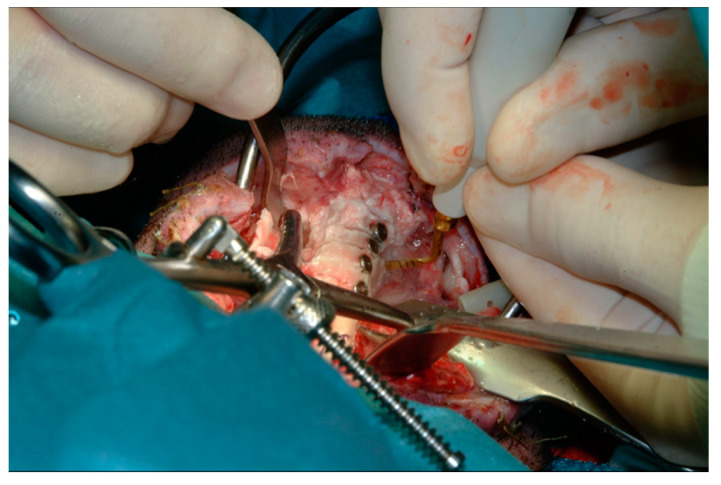
Sliced dissection using Piezosurgery.

**Figure 5:**
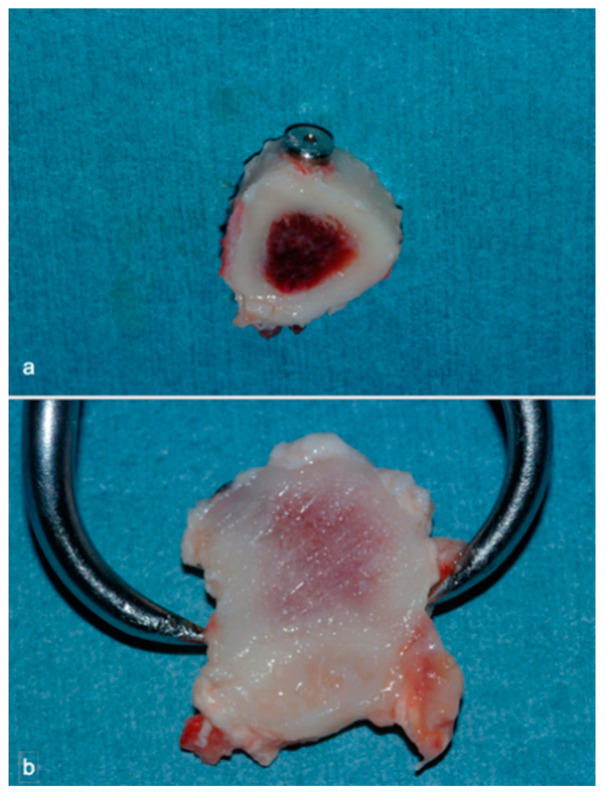
Bone sample including implant (**a**,**b**).

After 7, 14, and 56 days post-implant, animals were randomly euthanized by pre-anaesthesia with 2% xylazine (Rompun 2%, 2.3 mg/kg, Bayer S.p.A., Milan, Italy) (2.2 mg/kg) and tiletamine/zolazepam (Zoletil 100-Virbac 20%, 6.3 mg/kg, Laboratoires Virbac, Carros, France) (6.6 mg/kg), as well as an intracardiac injection of embutramide, mebezonium iodide, and tetracaine hydrochloride (Tanax: 70 mg/kg Intervent International GmbH, Unterschleißheim, Germany) (70 mg/kg). The tibias were exposed and dissected into slices to evaluate the peri-implant osseous healing corresponding to the various implant sites (Figure 4 and Figure 5). The volume of peri-implant bone used for analyses was consistent among all sample and was about three millimeters around each implant site.

### 2.3. Histological Analysis

The degree of inflammation, bone healing, and remodeling was evaluated at the Department of Medical Science, on bone samples obtained at 7, 14, and 56 days post-implant.

Once the implants had been removed, the specimens were fixed in 10% formalin for 24 h, decalcified for 3–4 days in a mixture of 50% formic acid and 10% sodium citrate tribasic, embedded in paraffin, sectioned along the longitudinal implant axis using a microtome (Electronic Motorized Microtome; Shandon-Lipshaw, Pittsburgh, PA, USA), and stained with hematoxylin-eosin for optical microscopy (DMLB Leica, Solms, Germany). The number of infiltrated inflammatory cells was evaluated for each case, as the number of polymorphonucleated and mononucleated inflammatory cells per high power field-mean of 10 fields (expressed as ratio on control taken as 1). The osteogenesis process as the number of osteoblasts per high power field-mean of 10 fields was expressed as a ratio on the control taken as 1 and observed at the magnification 100X.

### 2.4. Biomolecular Analysis

To avoid RNA degradation, all the specimens (7, 14, and 56 days) were placed in RNA Later solution (Qiagen, Milan, Italy) and stored at −80 °C until testing. The analyses were carried out by first grinding the specimens under a liquid nitrogen stream and extracting total RNA from the bone powder (150–200 mg), using the acid guanidinium thiocyanate-phenol-chloroform extraction method [20].

The RT-polymerase chain reaction (PCR) was performed using single-stranded cDNA prepared from total RNA (1 μg) with a High Capacity cDNA Archive kit (Applied Biosystems, Foster City, CA, USA). The forward (FW) and reverse (RV) primers were designed using the Beacon Designer software (Bio-Rad, Hercules, CA, USA) and are listed in Table 1.

### 2.5. Real-Time Polymerase Chain Reaction (PCR)

Twenty-five microliters of a PCR mixture, containing cDNA template equivalent to 80 ng of total RNA and 5 pmoles each of the forward and reverse primers and 2 × iQ^™^ SYBR® Green SuperMix (Bio-Rad, Hercules, CA, USA), were amplified using an iCycler PCR instrument (Bio-Rad, Hercules, CA, USA) to test for BMP-4, BMP-7, TGF-β2, Interleukin (IL)-1β and Osteocalcin (OC) or glyceraldehyde phosphate dehydrogenase (GAPDH, housekeeping gene). The PCR conditions were an initial melt at 95 °C for 3 min, followed by 45 cycles at 95 °C for 40 s, at 52 °C for 40 s, and at 72 °C for 40 s. Each sample was tested six times and the threshold cycle (Ct) values were averaged from each reaction. The fold change was defined as the relative expression compared to that at time 0, calculated as 2^−∆∆Ct^, where ∆Ct = Ct_sample_ − Ct_GAPDH_ and ∆∆Ct = ∆Ct_sample_ − ∆Ct_time 0_. All results were expressed in the Figures as ratio on control taken as 1.

Biomolecular analysis was performed at the Department of Clinical and Biological Sciences.

### 2.6. Outcome Assessor

The examiners did not know the specific implant site or from which animal the implants were taken or what type of protein had been used for that site.

To avoid biased results, only the dental surgeon knew the sites where the substances had been added and to which laboratory the samples were sent.

All circumstances during the intervention were similar in all animal groups.

All protocols and all pre-specified primary and secondary study outcomes are available and reported in the manuscript.

Baseline values of the outcomes are of interest in the study.

All primary outcomes reported using measurements, analysis methods, or data subsets, have been pre-specified in the protocol.

Each animal was subjected to the same protocol regarding anesthesia as well as surgical and housing procedures. The only difference was related to the different type of substance positioned in the implant site (rhBMP-7, Type 1 Collagen or FN).

The team consisted of a veterinarian anesthetist, two dental surgeon (one of them the lead researcher), a veterinarian surgeon, three pathologists (histological data analysis and biomolecular data analysis), and a veterinarian dedicated to animal logistics (who did not participate in the research).

Animals were sacrificed at the end of each experimental period regarding the methods and techniques. The study material was available post-mortem. All animal handling and housing was carried out in protected and dedicated environments in the Animal Pathology Department and in full compliance with the laws in force.

The risk of bias in individual studies was assessed using the ARRIVE and SYRCLE checklists [22,23].

The study has been free of contamination, and free of inappropriate influence of funders.

## 3. Results

Only four implants were placed in the tibia of each animal, for two reasons. First, to maintain a certain bone distance among the implants to exclude cross-reactions among the different implant sites, such as chemotaxis phenomena or effects of chemical mediators; second, to not excessively weaken the bone structure itself.

For this reason, control implants were placed only in the second animal group.

### 3.1. Histological Analysis

In sites prepared with FN or rhBMP-7, the peri-implant bone tissues showed inflammatory infiltration similar to the control sites, whereas the sites prepared with Type 1 Collagen induced a greater number of inflammatory cells than all other types of sites at 14–56 days.

In all samples, at 7 days, the inflammatory infiltrate was characterized by neutrophilic granulocytes, followed, at 14 and 56 days, by mononuclear cells (lymphocytes, plasma cells, and macrophages) in all samples.

Neo-osteogenesis was quantified considering the absolute number of osteoblasts per high power field (mean of 10 fields) in osteogenesis areas. Neo-osteogenesis was consistently more active in the samples prepared using addition of FN or rhBMP-7, compared to the control sites.

**Figure 6:**
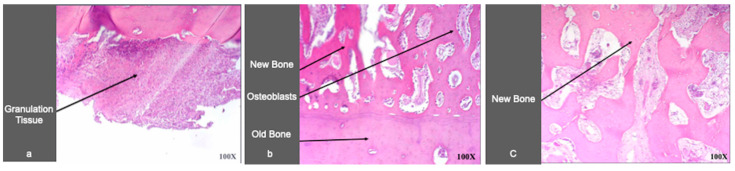
Examples of histological analysis in the presence of Fibronectin; 7 (**a**), 14 (**b**), and 56 (**c**) days (Hematoxylin and Eosin, 100X).

**Figure 7:**
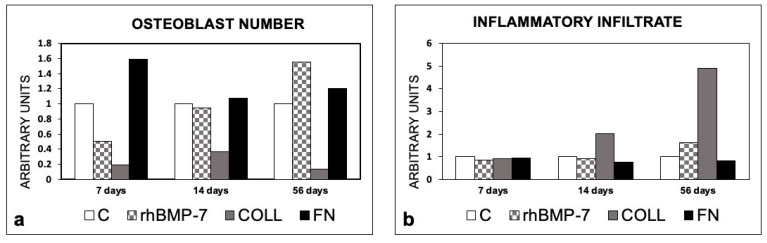
Osteogenesis and inflammatory process in peri-implant bone tissues from sites treated with rhBMP-7, Type 1 Collagen, or Fibronectin. (**a**) Osteogenesis was evaluated as the number of osteoblasts counted per high-power field (mean of 10 fields) and expressed as ratio of control sites taken as 1. (**b**) The inflammation process was evaluated as the number of neutrophilic granulocytes (7 days) and mononucleated (lymphocytes, plasma cells, macrophages, 14 and 56 days) inflammatory cells per high-power field (mean of 10 fields) and expressed as a ratio on control sites taken as 1. C, control sites; rhBMP-7, sites treated with rhBMP-7; COLL, sites treated with Type 1 Collagen; FN, sites treated with Fibronectin.

The addition of FN caused an increase of the osteoblasts already at 7 days in comparison to control sites; rhBMP-7 caused a higher increase of osteoblasts than in the controls at 56 days. On the contrary, the neo-osteogenesis was observed to be less active in the bone samples obtained from sites treated with of Type 1 Collagen in comparison with the control, FN, and BMP-7 (Figure 6 and Figure 7).

### 3.2. Biomolecular Analysis

#### 3.2.1. Bone Morphogenetic Proteins

An early (7 and 14 days) increase in the BMP-4 and 7 expression levels was observed in the sites that had been prepared with rhBMP-7and FN in comparison with the control site; at 56 days, a decrease in the expression of BMP-4 was observed in both sites. In rhBMP-7 and FN sites, the BMP-7 expression level was greater than in the control site at 56 days.

**Figure 8:**
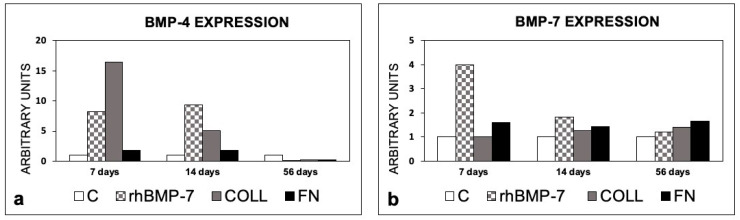
Expression of BMP-4 and BMP-7 in peri-implant bone tissues from sites treated with rhBMP-7, Type 1 Collagen, or Fibronectin. (**a**) BMP-4 expression; (**b**) BMP-7 expression. The values are expressed as a ratio on control sites taken as 1. C, control sites; rhBMP-7, sites treated with rhBMP-7; COLL, sites treated with Type 1 Collagen; FN, sites treated with Fibronectin.

In sites with Type 1 collagen alone, BPM-4 increased at 7 and 14 days, with values below those of the control at 56 days, while the expression of BMP-7 increased slightly from day 14 (Figure 8).

#### 3.2.2. Cytokines

**Figure 9:**
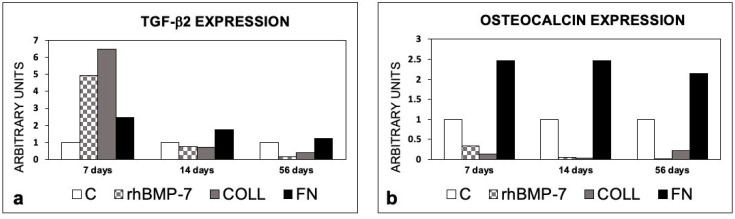
Expression of TGF-β2 and Osteocalcin in peri-implant bone tissues from sites treated with rhBMP-7, Type 1 Collagen, or Fibronectin. (**a**) TGF-β2 expression; (**b**) Osteocalcin expression. The values are expressed as a ratio on control sites taken as 1. C, control sites; rhBMP-7, sites treated with rhBMP-7; COLL, sites treated with Type 1 Collagen; FN, sites treated with Fibronectin.

At all experimental times, an increase in the TGF-β2 level was observed in sites prepared with FN compared to the control sites, whereas the increase of the level of TGF-β2 was observed only at 7 days in the presence of rhBMP-7 (Figure 9).

**Figure 10:**
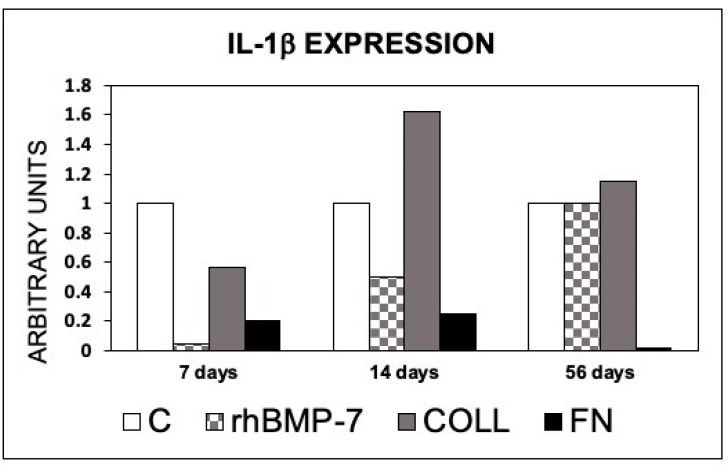
Expression of IL-1β in peri-implant bone tissues from sites treated with rhBMP-7, Type 1 Collagen, or Fbronectin. The values are expressed as a ratio on control sites taken as 1. C, control sites; rhBMP-7, sites treated with rhBMP-7; COLL, sites treated with Type 1 Collagen; FN, sites treated with Fibronectin.

An increase in the IL-1β mRNA was observed in the sites treated with Type 1 Collagen at 14 and 56 days compared to the control site. No increase in IL-1β was observed in the other sites, compared to the control site (Figure 10).

#### 3.2.3. Osteocalcin

Figure 9 reports the expression of OC, which was higher only in F-treated sites in comparison with the control and other sites, irrespective of the time.

## 4. Discussion

To evaluate the osseointegration of dental implants added with rhBMP-7, Type 1 Collagen, or FN, bone samples were collected at different experimental times (after 0, 7, 14, and 56 days).

Time 0 corresponded to the baseline values present in the bone taken, from each animal, at the time of implant positioning; 7 and 14 days were chosen because they represent the early stages of the osseointegration process; 56 days was chosen because it has been demonstrated that at this time mature bone is already present around porous implants [1].

The BMP family belongs to the TGF-β superfamily. In particular, BMP-2, BMP-4, and BMP-7 are the most important responsible for bone and cartilage production. Differently, BMP-3 and BMP-13 counteract bone deposition [24,25,26,27].

Based on their osteogenic properties, BMPs have been studied for clinical applications in different medical fields. In particular, rhBMPs have been produced and used in various types of pathologies, evidencing both positive and side effects.

Retrospective studies evidenced that rhBMP-2 and -7 induced an early and transient bone resorption due to the osteoclast stimulation and that new-osteogenesis leading to bone repair occurred subsequently [28,29,30,31].

An important aspect of the healing process is the inflammatory phase, which precedes and favors tissue regeneration/repair. Anyway, to avoid tissue damage, it is important that inflammation is not redundant.

For this reason, in this study, the entity of the inflammatory process was evaluated by counting neutrophilic granulocytes and mononuclear cells present in bone surrounding implants.

As far as we know, the present study is one of the few research works that have investigated in vivo in minipigs both the histological events and the molecular pathways occurring after positioning of dental implants in “clinical use”.

The experimental design was based on the previous experience of the authors with this animal model, in which the anatomical characteristics of the tibia are similar to those of the human mandibular bone with regard to the cortical/cancellous relationship and the bone quality, as can be seen in Figure 1 [1,10,11].

The tibial anatomical region was also chosen for the considerable difficulty in placing implants in the oral cavity of these animals [32].

### 4.1. rhBMP-7, Type 1 Collagen and Fibronectin Effects on Osteogenesis and Inflammation Process

#### 4.1.1. rhBMP-7

Even if rhBMP-7 has been previously demonstrated to facilitate the healing process of long bones [33], few studies have investigated its effect in dentistry and, in particular, in dental implants, where mainly rhBMP-2 has been studied [34,35,36,37].

In oral tissue engineering, rhBMPs have been shown to stimulate pulp stem cells to differentiate into odontoblasts, facilitating pulp and dentin regeneration and to improve implant osseointegration [31,38,39,40,41,42].

The absorption of human BMP-7 to titanium discs coated with poly-ethyl acrylate (PEA) induced the differentiation of the mesenchymal cells into osteoblasts and the biological activity of titanium implants with BMP-7 alone, reducing the administered dose [43].

In different animal models, a major bone production has been observed in BMP-7-treated implants [17,28,44,45].

In 2010 and 2017, systematic reviews analyzed the effects of BMPs on bone production after dental implant positioning and concluded that the results, even if positive, were not sufficient to definitively confirm their applications in dentistry [46,47].

In the present study, in rhBMP-7 treated implant sites, an early decrease in osteoblast number has been observed in comparison with the control site, followed by an increase and by a maximum at 56 days (1.55 vs. controls). The decrease at 7 days could still be due to the surgery trauma and the presence of the granulation tissue, as can be seen in Figure 6.

The increase in osteoblast number in this experimental model confirms, in agreement with previous studies, the ability of rhBMP-7 to improve the deposition of peri-implant bone [17,28,39,40,41,42,43,44,45,48].

Moreover, the sites treated with rhBMP-7 showed, at 56 days, an increase in the inflammatory infiltrate compare to the control site values; IL-1β (pro-inflammatory protein) never exceeded the control sites values.

This result could be related either to the BMP dose administered (as previously highlighted, the dose can modulate the effects [49]) or to the collagen carrier transporting the rhBMP-7 [24,32,50]. In any case, the inflammatory process seems to be controlled because the number of osteoblasts is higher than in the control site and the IL-1β (pro-inflammatory protein) is similar to that in the control site.

#### 4.1.2. Collagen

We analyzed the effect of Type 1 Collagen alone since this molecule is used as carrier for BMPs, even if at the present, the gold-standard carrier has not been yet identified and it is possible that it varies in relation to specific clinical applications [51,52,53,54,55].

However, the geometry of the carrier can also significantly affect capillary penetration [56]; moreover, it cannot be excluded that the optimal profile may also be site-specific [57]. For these reasons, several materials have been investigated, including: natural polymers and synthetic polymers, inorganic materials and their composites [55,58,59,60,61].

Animal studies have documented that BMPs, carried by a collagen vector, favoured the deposition of new endochondral bone in several animal species [62,63,64].

However, although many substances have been evaluated as transporters, collagen is the only BMPs carrier approved by the Food and Drug Administration (FDA) for clinical use in orthopedics [57,61,65].

In the present study, in implant sites treated with Type 1 Collagen alone, an increased number of inflammatory cells was evidenced towards the end of the experimental period, coupled with a corresponding increase in pro-inflammatory IL-β1 protein. The observed induction of a late inflammatory process could be responsible for the reduced osteogenesis, attested by the low number of osteoblasts present. Despite the induction of inflammation and the low bone production, no loss of implants occurred in Type 1 Collagen-treated implant sites. This observation disagrees with the results of Stadlinger et al. who evidenced an immune-mediated failure of implants positioned in the presence of collagen alone [32].

#### 4.1.3. Fibronectin

Extracellular adhesion proteins, such as vitronectin and FN, favoring cell adhesion, proliferation, and differentiation via interaction with specific integrins, play a pivotal role in implant osseointegration [66,67].

Unfolded FN has also been shown to act as a binding site for many growth factors, including BMP-2, with this favoring mesenchymal stem cell differentiation [68].

In the present study, the number of osteoblasts in the bone surrounding FN-treated implants was higher than that observed in control implants at all the experimental times, with a major increase at 7 days after surgery.

This result confirms the reported ability of FN to facilitate early osteoblast adhesion and differentiation and agrees with previous observations in vitro on human and murine osteoblast-like cells, and in vivo after positioning of FN-modified dental titanium implants in dogs [66,69,70].

In the case of FN-treated implants, the number of inflammatory cells was similar to that observed in control sites at 7 days and decreased towards the end of the experimental period, indicating that no prolonged and inappropriate induction of phlogosis occurs in the presence of FN. The ability of FN to reduce inflammation was confirmed by the decreased expression of IL-1β at all experimental times.

Up to now, few research works have investigated the pro- or anti-inflammatory properties of FN, and the reported results are contradictory, mainly in relation to the experimental protocol used.

Our results agree with those of Li et al. [71], who evidenced that a heparin/fibronectin complex immobilized onto a titanium surface early decreases the number of macrophages and their responsivity to TNFα, a well-known pro-inflammatory molecule. Moreover, a decrease in IL-1β release has also been observed in heparin/fibronectin-treated implants.

A similar anti-inflammatory effect has been reported in the case of monocyte-derived macrophages seeded on FN-coated poly (L-lactic acid) films, where a significant reduction in IL-6 (inflammatory cytokine) release and an increase in IL-10 (anti-inflammatory protein) were observed [72]. Differently, expanded polytetrafluoroethylene treated with FN has been shown to induce an extensive inflammatory process when inserted into the adipose tissue of rats. In this case, foreign body giant cells typical of chronic inflammation have been also observed [73].

### 4.2. Expression of BMP-4, BMP-7, TGF-β2, and Osteocalcin in Implant Sites Treated with rhBMP-7, Type 1 Collagen and Fibronectin

During the formation of bone after a fracture, the expression of BMP-4 increases very early and is involved in the recruitment of mesenchymal stem cells and the inhibition of osteoclast activity, thus facilitating new bone production; moreover, its ability to stimulate chondrogenesis BMP-4 is crucial in inducing enchondral ossification [74,75].

In contrast to BMPs, TGF-β family members are not able to stimulate bone formation in ectopic sites but increase osteoid deposition and mineralization when directly added to bone.

As BMPs, TGF-β also inhibits osteoclast function by inducing apoptosis in differentiated ones [76]. Moreover, the anti-inflammatory properties of TGF-β2 have been extensively documented [77,78,79].

In dentistry, it has been reported that TGF-β2 improves bone regeneration around the implants [80].

Osteocalcin, the most present non-collagenous protein in bone, is usually considered a marker of osteoblast functionality and has been recently indicated as important, mainly for the alignment of apatite crystals [81].

In the present pilot study, rhBMP-7 and Type 1 Collagen alone modulated, in a similar way, the expression of all the molecules examined (BMP-4, BMP-7, TGF-β2, and OC).

At 7 days, Type 1 Collagen caused a major increase in the expression of BMP-4 and TGF-β2. The ability of collagen in early increasing these osteogenic factors agrees with the results of a previous study [82] but does not correspond to an increased number of osteoblasts.

The latter observation confirms the previous results of Shi et al. [83], suggesting that collagen is essential, but not sufficient to induce the complete differentiation of Mesenchymal Stem Cells (MSC) into osteoblasts.

However, animal studies have shown an increase in peri-implant bone density, in sites with implants coated with collagen alone [84,85].

The contradictory results present in the literature in terms of collagen suggest that the effects triggered by this molecule are likely influenced by several factors.

The low expression of BMP-4 and TGF-β2 at 56 days in the sites treated with rhBMP-7 and Type 1 Collagen is due to the early involvement of these proteins in bone formation [10,86,87].

The expression of BMP-7 was increased by rhBMP-7 at all experimental times. Type 1 collagen induced BMP-7 at 14 and 56 only. The authors are unable to explain the low BMP-7 expression, at 7 days, in the sites treated with collagen alone.

The set of effects evidenced by rhBMP-7 confirms its ability in favoring osteoblastic differentiation and implant osseointegration and agrees with the results obtained by Kirkwood et al. [88] with BMP-7 immobilized to titanium implants.

The addition of both rhBMP-7 and Type 1 Collagen alone decreased the OC expression at all experimental times, which is in agreement with a previous study [42] reporting a low expression of OC in human MSC grown for 28 days on titanium discs coated with poly (ethyl acrylate) and adsorbed with BMP-7.

Furthermore, the expression of OC seems to be stimulated more by BMP-2 than by BMP-7 [89].

Several studies reported that BMP-7 decreases the expression of FN in clinical and experimental pathological conditions [90,91,92]. However, the ability of FN to modulate BMP-7 expression has not yet been investigated.

In the current study, FN addition increased the mRNA level of BMP-7 at all the experimental times, suggesting that the osteogenic properties of FN are also mediated by its ability to induce the expression of this BMP, which plays an important role in osteoblast differentiation. 

In a similar way, in FN-treated sites the expression of BMP-4 was increased at 7 and 14 days; the drastic decrease observed at the last experimental time point could be related to the fact that BMP-4 is a major driver of early phases of osteogenic processes, as previously reported [10,86,87].

Among cytokines, the TGF-β family regulates numerous cell functions, is involved in the maintenance of bone homeostasis, and shows a different expression time course during fracture healing. In particular, TGF-β2 and 3 display the highest production at 7 days after the damage [79].

The results of the present study agree with this observation. In fact, in FN-treated sites, the level of TGF-β2 mRNA was always higher than in the control site, with a major increase already present at 7 days. 

The ability of FN addition to induce osteoblast activity, other than to increase their number, is evidenced by the high expression of OC observed at all experimental times.

### 4.3. Histological Analysis

Histological and histomorphometric analyses represent a good method to evaluate spatial and temporal events occurring during osseointegration of dental implants [28,45].

In the present study, the results of the evaluation of osteoblast number and inflammatory infiltrate in the different implant sites completely corresponded to those obtained by biomolecular analyses, confirming that bone formation was always higher in sites treated with rhBMP-7 and FN and coupled with a reduced induction of inflammatory processes.

### 4.4. Limitations of the Study

The study presents some limitations.

1. Since the report was a pilot study, for each experimental time, both histological and biomolecular analyses were carried out only in one site, generating a statistical problem.
  In fact, although it is possible to identify a trend, the sample number was reduced to apply a statistical model. Although each sample, for biomolecular analysis, was tested six time, for histological analysis, the inflammatory cells and osteoblasts were tested in 10 fields.
2. It would have been good to investigate more anti-inflammatory proteins.
3. If the study was to be redone, hetero-dimers would probably have to be used as they are less expensive. This could be the starting point for a future study. However, it must be kept in mind that hetero-dimers are the combination of more BMPs, reducing the possibility in distinguishing the effect of a single BMP type.
4. Although the animal models are accepted as a standard in the study of dental implants, some obstacles can arise. The results obtained are not mathematically exportable to human beings. However, the experimentation of these proteins in vivo on humans, as regards to dental implants, is currently highly difficult in terms of authorization, at least in Italy.

### 4.5. Values of the Study

This report adds to the few data present in the literature in terms of biomolecular pathways triggered by exogenous addition of factors able to improve osseointegration of dental implants.

The study design investigates, in the same implant site, the expression of some factors involved in bone production and inflammation induction. The effect of Type 1 Collagen alone was also evaluated. Moreover, the biomolecular analysis is coupled with histological examination. All of this is a rare case in a scientific study evaluating the osseointegration of dental implants.

## 5. Conclusions

1. Collectively, the reported results of this initial pilot study evidence, in a novelty way, that FN and rhBMP-7 addition during dental implant positioning in animals is able to early modulate the expression of cytokines belonging to the TGF-β superfamily (BMP-4, BMP-7, TGF-β2), suggesting that these signal transduction pathways are modulated by these proteins and contribute to improving the neo-osteogenesis and the osseointegration process of implants.
2. Type 1 Collagen induces inflammatory infiltrate in the peri-implant tissues. It is not able to induce bone formation, although at 7 days, it increased the local expression of BMP-4 and TGF-β2.
  However, these results will need to be confirmed in future studies conducted on a larger sample to endorse the findings obtained.

### Concluding Remarks

Although the current results in using Bone Morphogenetic Proteins (BMPs) seem attractive, some significant problems are yet unsolved.

It has been suggested that the administration method, the formulation features, or the instability of the BMPs could cause some clinical side effects.

In fact, it has recently been shown that inflammation process and pain could be related to the non-optimal method of administration and protein instability.

Moreover, the healing mechanisms can be negatively affected by intra- or extracellular inhibitors of BMPs that are overexpressed after BMP administration.

The knowledge of interactions between BMPs and their inhibitors could represent a crucial point to improve the efficacy of these proteins.

In a similar way, few studies have compared the effects of various types of vectors on the quantity and quality of formed bone.

Another important aspect that has yet to be fully clarified is the difficulty of reproducing the positive results obtained in animal models in humans, maybe for a species-specific response or for a non-optimal dosage.

Therefore, despite the significant evidence of bone healing potential shown by BMPs in animal models, clinical studies will have to be performed to better understand the importance of variables, such as dose, carrier, and administration way.

Nevertheless, in recent decades, some progress has been made in understanding the relationship among protein configuration and its effectiveness and safeness, besides the interaction with the environment in which it is placed.

Regarding the use of BMPs in dental implantology, to date, there are few randomized clinical trials to establish clinical protocols to increase bone volume before implant placement or to improve the implant osseointegration process.

The difficulty in comparing the results of the use of growth factors in dental implantology is due to the multiple differences in the study protocols, the use of different concentrations of growth factors, and of various types of vectors.

Future studies need to investigate these aspects to obtain information important to further improve the conditions of use of BMPs, thereby increasing their effectiveness and reducing their side effects.

Against this background, BMPs and other less known morphogens could be used in a much wider range of clinical conditions, in oral and maxillofacial surgery bringing great clinical benefit not only in oral implantology, but above all in post-traumatic, iatrogenic, or post-neoplastic bone regeneration.

## Figures and Tables

**Table 1:** Primer sequences for PCR.

Gene	Accession Number	Primer Sequences
BMP-4 (SUS SCROFA)	[21]	FW 5′-CTCGCTCTATGTGGACTTC-3′ RV 5′-ATGGTTGGTTGAGTTGAGG-3′
TGF-β2 (HOMO SAPIENS)	AY438979	FW 5′-GAGACTTGATTGTCCTTCCTTC-3′ RV 5′-CTCCCCGAACCGTTGAGG-3′
IL-1β (SUS SCROFA)	NM_001005149	FW 5′-GGGGACTTGAAGAGAGAA-3′ RV 5′-CATCACACAAGACAGGTACAGA-3′
OSTEOCALCIN (SUS SCROFA)	AY150038	FW 5′-TATGGCATAGCCTAGACCTC-3′ RV 5′-GATGATGGGGACCTTACACTT-3′
BMP-7 (HOMO SAPIENS)	NM_001719	FW 5′-GTGGAACATGACAAGGAAT-3′ RV 5′-GAAAGATCAAACCGGAAC-3′
GAPDH (HOMO SAPIENS)	NM_002046	FW 5′-TGAAGGTCGGAGTCAACGGATTTGGT-3′ RV 5′-CATGTGGGCCATGAGGTCCACCAC-3′

BMP-4, Bone Morphogenetic Protein-4; TGF-β2, Transforming Growth Factor-β2; IL-1β, Interleukin-1β; OC, Osteocalcin; BMP-7, Bone Morphogenetic Protein-7; GAPDH, glyceraldehyde 3-phosphate dehydrogenase.

## Data Availability

The animals were used as there are no alternatives for this type of study. The number of animals used was reduced to a minimum. In all the experimental phases, protocols were applied to eliminate any type of discomfort or pain to the animals.
